# Analytical Method Development and Dermal Absorption of Pyrogallol, a Hair Dye Ingredient

**DOI:** 10.3390/toxics10100570

**Published:** 2022-09-29

**Authors:** Yu-Jin Kim, Hyang-Yeon Kim, Jung-Dae Lee, Hong-Yoon Kim, Jueng-Eun Im, Kyu-Bong Kim

**Affiliations:** 1 College of Pharmacy, Dankook University, 119 Dandae-ro, Cheonan 31116, Chungnam, Korea; 2 Center for Human Risk Assessment, Dankook University, Cheonan 31116, Chungnam, Korea; 3 Medical AI Research Team, Chungbuk National University Hospital, Cheongju 28644, Chungbuk, Korea

**Keywords:** Franz diffusion cell, high-performance liquid chromatography (HPLC)

## Abstract

Pyrogallol is an ingredient in hair dye. Its concentration in hair dye is managed at less than 2.0% in Korea. There have been no reports on the dermal absorption rate of pyrogallol. The two purposes of this study were to develop an analytical method and determine the dermal absorption rate of pyrogallol. An analytical method was developed and validated by high-performance liquid chromatography (HPLC) analysis of various matrices including swabs (SWAB), skin (SKIN, dermis + epidermis), stratum corneum (SC), and receptor fluid (RF). Linearity (r^2^ = 0.9993–0.9998), accuracy (92.1–108.2%), and precision (0.5–9.5%) met the validation criteria in guidelines. A Franz diffusion cell was used to determine the dermal absorption of pyrogallol using the skin of mini pigs. Pyrogallol (2.0%) was applied to the skin (10 μL/cm^2^). For the actual hair dye conditions, the skin was wiped with a swab 30 min after application. Twenty-four hours later, it was wiped with a swab again and the SC was collected using tape stripping. All samples were extracted with water and analyzed. RF was recovered at 0, 1, 2, 4, 8, 12, and 24 h. The total dermal absorption rate of pyrogallol was determined to be 26.0 ± 3.9%.

## 1. Introduction

Hair dye is composed of several ingredients, one of which is pyrogallol. Pyrogallol is a white powder with a molecular weight of 126.11 g/mol. Pyrogallol has an XLogP3 value of 0.5, and 1 g dissolves in 1.7 mL of water [1]. The detailed physicochemical properties of pyrogallol are shown in Table 1. Pyrogallol is naturally present in plants as a decomposition product of hydrolyzable tannins [2]. It is a reducing agent that absorbs oxygen from the air in an alkaline solution and becomes darker when oxidized. Due to its oxygen radical-generating property, pyrogallol is commonly used in photographic agents and the dyeing industry [2]. It was the first synthetic organic dye used on human hair [3]. Pyrogallol is widely used not only in the cosmetic industry, but also in pharmaceuticals, agriculture, and food [4]. However, the cosmetic industry (hair dye) is increasingly trying to ban its use due to its toxicity [5]. In Korea and Japan, the concentration of pyrogallol in hair dyes is managed at less than 2.0% [6,7]. However, its use as a raw material for dyes is prohibited in Europe [8]. Hair dyes sold in the United States in the late 1980s and early 1990s contained 0.1 to 5.0% pyrogallol. However, pyrogallol-based hair dyes are not currently available to the public [5].

According to the ECHA (European Chemicals Agency), pyrogallol is hazardous when exposed through oral, skin, and inhalation routes. Pyrogallol has been reported to show genotoxicity and skin sensitization [9,10,11]. The genotoxicity of pyrogallol was analyzed by the bacterial reverse mutation test (Ames test) using Salmonella typhimurium TA1535, TA1537, TA1538, TA98, and TA100 strains [9]. In the absence of S9 metabolic activation, pyrogallol induced mutations in strain TA1537. Additionally, in the TA98 and TA100 strains, pyrogallol induced mutations with and without S9 metabolic activation [9]. In the mouse micronucleus test, pyrogallol was intraperitoneally administered at doses of 0, 68, 126, and 252 mg/kg. Compared to the control group, the proportion of micronucleated polynuclear erythrocytes was significantly increased in the 126 and 252 mg/kg dose groups [9,10].

Considering skin sensitization, a study in female BALB/c mice confirmed contact hypersensitivity when pyrogallol was applied to the skin. Pyrogallol was applied at concentrations of 0.25, 0.5, 1, 2.5, 5, 10, 25, and 50% in a skin sensitization study using the local lymph node assay (LLNA). A significant increase in lymph node cells was observed at treatment concentrations above 0.5% pyrogallol. These results suggested that pyrogallol is a sensitizer. To support these results, a mouse ear edema test was additionally performed. After 72 h of application of 0.5% pyrogallol, the thickness of the mice ears increased, indicating that pyrogallol is a skin sensitizer in BALB/c mice [10,11]. In another test, natural pyrogallol and synthetic pyrogallol were applied to the skin of the back of female guinea pigs. Patches were applied for 24 h and reactions were graded according to the set time. Slight erythema was observed at the sites treated with natural pyrogallol and synthetic pyrogallol and after removing the patch, the skin was dry and thickened. Thus, these two substances were classified as slight sensitizers to the skin of guinea pigs [3].

However, no data on the dermal absorption of pyrogallol, which is known to cause skin sensitization, have been reported. This study developed a new analytical method for pyrogallol and the dermal absorption of pyrogallol was determined using the method. The calculated dermal absorption rate could be used as a parameter for the further risk assessment of pyrogallol exposure.

## 2. Materials and Methods

### 2.1. Chemicals

Pyrogallol was purchased from Tokyo Chemical Industry Co. (Tokyo, Japan). Phosphate-buffered saline (PBS) and hydrogen peroxide solution were purchased from Sigma Aldrich Co. (St. Louis, MO, USA). Distilled water (DW), acetonitrile (ACN), and methanol were purchased from Honeywell Burdick & Jackson Co. (St. Harvey, MI, USA). Hydrogen peroxide was purchased from Sigma Aldrich Co. (St. Louis, MO, USA).

### 2.2. HPLC Condition

High-performance liquid chromatography (HPLC) analysis of pyrogallol was performed using an Agilent 1290 Infinity LC (Agilent Technology, Waldbronn, Germany). Samples were analyzed by ultraviolet-visible spectroscopy (UV-Vis) detection at a wavelength of 200 nm. A Luna C18 (150 × 3.00 mm i.d. 3 μm) (Phenomenex, CA, USA) column with Security Guard Cartridges RP-1 (4 × 3.0 mm; Phenomenex) was used for pyrogallol analysis. Analysis was performed with isocratic conditions and a mobile phase consisting of 0.1% formic acid in DW (solvent A) and ACN (solvent B). The flow rate and injection volume were 0.35 mL/min and 10 μL, respectively. The detailed HPLC conditions for pyrogallol are shown in Table 2.

### 2.3. Validation of Pyrogallol

#### 2.3.1. Calibration Standards and QC Sample

Calibration solutions were made by mixing 15 μL of standard solution into 135 μL of blank swab (SWAB), stratum corneum (SC), skin (SKIN), and receptor fluid (RF) matrices. Since pyrogallol is soluble in water, a stock concentration of 10 mg/mL was prepared in DW. The standard solution was diluted with the stock solution to prepare 5, 10, 20, 50, 100, and 200 μg/mL concentrations. After 10-fold dilution, the final concentrations of the calibration standards were 0.5, 1, 2, 5, 10, and 20 μg/mL. The lower limit of quantitation (LLOQ) of the quality control (QC) sample was determined to be 0.5 μg/mL. The limit of quantitation (LOQ, 1.5 μg/mL), minimum of quantitation (MOQ, 7.5 μg/mL), and high of quantitation (HOQ, 15 μg/mL) were determined. To extract pyrogallol from the calibration solutions, 50 μL of each calibration solution was mixed with 200 μL of 50% methanol. The mixtures were centrifuged at 15,928× *g* for 10 min and the supernatant was filtered through a 0.2 μm polytetrafluoroethylene (PTFE) filter (ADVANTEC, Dublin, CA, USA).

#### 2.3.2. Accuracy and Precision

Accuracy and precision were determined in accordance with the guidelines for validating bio-sampling methods of the Korea Ministry of Food and Drug Safety [12]. In the intra-day tests, samples were repeated five times on the same day, and in the inter-day tests, samples were repeated three times on three days. Accuracy is a measure of the proximity of an experimental value to the actual amount of a substance in a matrix [13]. Accuracy was calculated by dividing the measured concentration by the nominal concentration and multiplying by 100. Precision was calculated as the coefficient of variation (CV). CV is the standard deviation divided by the mean. According to guidelines, accuracy and precision should be within 20% of the LLOQ concentration and within 15% of the LOQ, MOQ, and HOQ values [12].

### 2.4. In Vitro Dermal Absorption

This study was conducted in accordance with in vitro skin absorption test guidelines [14,15]. The assay was performed with Franz diffusion cells using six minipig skins with a thickness of 500 μm (Apures, Pyeongtaek, Korea). Because pyrogallol is a water-soluble substance, the RF used was 0.01 M PBS. According to the Korean hair dye management standards [6], 4.0% pyrogallol and 6.0% hydrogen peroxide were mixed at a 1:1 ratio to make 2.0% pyrogallol. The final concentration of 2.0% pyrogallol was applied to the skin of mini pigs at 10 μL per 1 cm^2^. The minipig skin was wiped with an alcohol swab after 30 min according to the actual hair dye usage conditions. Additionally, after 24 h, it was wiped again with an alcohol swab. Then, the SC of the skin was collected using tape stripping. The tape (ScotchTM, 3M, Maplewood, MN, USA) was cut into 1.5 × 1.5 cm-sized pieces and removed 15 times. Then, the minipig skin was cut into 8 pieces. The SWAB (30 min and 24 h), SC, and SKIN samples used in each step were put into 10 mL of water, sonicated for 1 h, and refrigerated for 24 h prior to analysis. RF was collected at 0, 1, 2, 4, 8, 12, and 24 h, and stored refrigerated prior to analysis.

### 2.5. Analysis Preparation

Extracted samples (SWAB, SC, SKIN) and RF were required each 50 μL. The extracted samples and RF were mixed with 200 μL of 50% methanol, centrifuged at 15,928× *g* for 10 min, and the supernatant was filtered through a 0.2 μm PTFE filter (ADVANTEC). Analysis was carried out under the HPLC conditions mentioned above.

## 3. Results

### 3.1. Linearity of Calibration Standards

**Figure 1:**
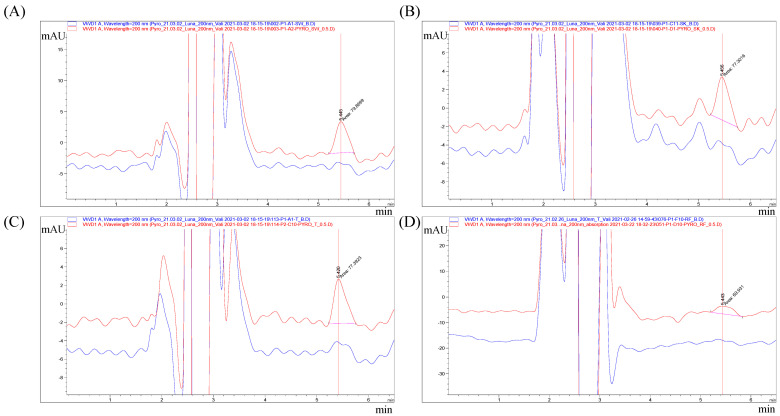
Chromatograms of pyrogallol at the blank, LLOQ (0.5 μg/mL) in SWAB (**A**), SKIN: dermis + epidermis (**B**), SC: stratum corneum (**C**), and RF: receptor fluid (PBS) (**D**). Blue line: blank; red line: LLOQ (0.5 μg/mL).

Calibration solutions were prepared at concentrations of 0.5, 1, 2, 5, 10, and 20 μg/mL. The chromatograms of blank and the LLOQ for SWAB, SC, SKIN, and RF samples are shown in Figure 1. The retention time of pyrogallol was 5.4 min. The FDA guidelines for validating analytical procedures recommend that r^2^ be submitted when evaluating linear relationships [16,17]. The slope should show a clear correlation between the response and analyte concentration. The results should not show large deviations from linearity, which is considered to imply a correlation coefficient of r^2^ > 0.99 [18]. The r^2^ values for the SWAB (30 min and 24 h), SC, SKIN, and RF samples were 0.99987, 0.99948, 0.99953, and 0.99939, respectively, showing good linearity of the calibration curve of pyrogallol in SWAB, SC, SKIN, and RF samples.

### 3.2. Accuracy and Precision

The mean accuracy of the SWAB (30 min and 24 h) sample determinations was 99.9–103.9% and the precision was determined to be 0.5–4.9% based on the CV. The mean accuracy of the SC sample determinations was 100.7–106.1% and the precision was determined to be 0.8–3.3% based on the CV. The mean accuracy of the SKIN sample deter-minations was 99.2–104.9% and the precision was determined to be 0.8–3.3% based on the CV. The mean accuracy of the RF sample determinations was 92.1–110.1% and the precision was determined to be 1.1–14.1% based on the CV. The intra-day and inter-day accuracy and precision met all criteria suggested by the guidelines [12]. The detailed intra-day and inter-day validation results are shown in Table 3.

### 3.3. In Vitro Dermal Absorption Experiment

**Figure 2:**
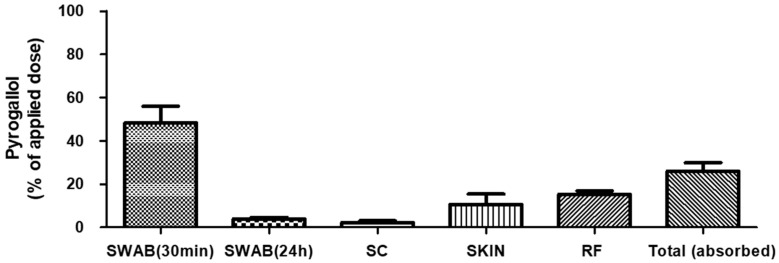
In vitro dermal absorption rate of pyrogallol. SKIN: dermis + epidermis, SC: stratum corneum, and RF: receptor fluid (PBS).

The recovery of pyrogallol was 48.4 ± 7.6% (mean ± standard deviation) for 30 min SWAB samples, 3.9 ± 0.8% for 24 h SWAB samples, 2.3 ± 0.7% for SC samples, 10.7 ± 4.7% for SKIN samples, and 15.3 ± 1.6% for RF samples. The total recovery was 80.5 ± 6.8% and the total absorption was 26.0 ± 3.9% (91.9 ± 13.7 μg/cm^2^). The detailed results are shown in Table 4 and Figure 2.

### 3.4. Permeation Parameter

**Figure 3:**
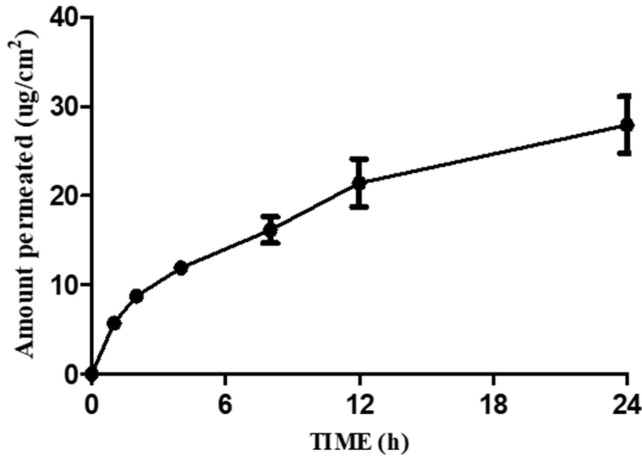
Permeation profiles of pyrogallol through excised mini pig skin.

Steady-state permeate flux (Js) and the permeability coefficient (Kp) serve as key parameters evaluated in in vitro experiments in which the donor concentration of the permeabilizing agent is maintained at constant dose conditions [19]. Permeation values were calculated from RF concentrations measured as cumulative pyrogallol of receptors at each sampling point normalized to the exposed skin surface area (1.77 cm^2^). Experiments and calculations have been carried out in this way when testing other materials, and this experiment was conducted in the same way [20]. The Js value was estimated to be 1.27 ± 0.13 μg/cm^2^/h (Table 5). The cumulative amount of pyrogallol reached in RF after applying 2.0% pyrogallol is shown in Figure 3.

## 4. Discussion

A London chemist and a Parisian hairdresser introduced 3% hydrogen peroxide to the hair industry, which led to the development of modern chemical hair colorants, and more dyes began to be produced in the late 19th century [21]. The first synthetic organic hair dye developed was pyrogallol, a substance naturally occurring in walnut shells. Since 1845, pyrogallol has been used to dye hair brown and is often combined with henna [21]. Synthetic compounds such as para-phenylenediamine, para-toluene diamine, and pyrogallol form quinone di-imine, and are known as coloring agents when combined with hydrogen peroxide [22].

Prior to the in vitro dermal absorption study, the HPLC analysis method for pyrogallol was validated. According to the guidelines, the accuracy and precision should be within 20% of the LLOQ concentration and within 15% of the LOQ, MOQ, and HOQ values [12]. Overall, the accuracy and precision of the QC sample for all concentrations of the sample satisfied the Ministry of Food and Drug Safety (MFDS) guidelines.

This study showed the dermal absorption rate of pyrogallol using a Franz diffusion cell, which has been frequently used for in vitro dermal absorption studies [23]. In order to proceed in the same way as the actual hair dye, the washing process was carried out after 30 min, and most of the exposed hair dye (48.4 ± 7.6%) was found in the washing solution (30 min). The total dermal absorption rate of pyrogallol for 24 h was 26.0 ± 3.9%.

Similar structural and physicochemical properties are expected to have similar dermal absorption properties [24]. Pyrocatechol (catechol), which has a structure similar to pyrogallol, is also used as a hair dye ingredient and is also known to be an irritant to the skin of albino rabbits. In the in vitro dermal absorption test, 64 μL of radiolabeled pyrocatechol (area 0.64 cm^2^) was applied to human skin [25]. The results of short-term experiments showed that catechol penetrated through human skin membranes to a very low degree within the first 1 h of exposure. An average of 26.65% penetrated human skin after 24 h of exposure [25]. After a delay of 6 h, pyrocatechol penetrated through the human skin membrane with a flux of 1.425 μg/cm^2^/h (permeation rate at steady-state). When comparing the dermal absorption rates of the two substances over a 24 h period, pyrogallol and pyrocatechol showed similar dermal absorption rates [25]. Factors affecting the dermal absorption rate are the octanol/water partition coefficient (LogP) and molecular size [24]. Pyrocatechol (catechol), which has a structure similar to pyrogallol, has a molecular weight of 110.11 g/mol, similar to pyrogallol, and LogP is also similar to 0.9 [25]. Currently, a skin absorption model is constructed and predicted with QSAR in consideration of similar physicochemical properties (molecular weight, LogP), exposure pathways, and conditions [25].

Dermal absorption can be an important factor for systemic toxicity, and the determination of the effective absorption amount is essential for human risk assessment [26]. The dermal absorption rate of pyrogallol determined in this study could be used as a parameter for the risk assessment of pyrogallol exposure.

## 5. Conclusions

The validation of the pyrogallol analytical method demonstrated that the developed HPLC method was a useful and rapid technique for the separation and quantitation of pyrogallol in samples (SWAB, SC, SKIN, and RF). The dermal absorption rate of pyrogallol, a hair dye ingredient, was 26.0 ± 3.9% (91.9 ± 13.7 μg/cm^2^).

## Figures and Tables

**Table 1:** Physicochemical properties of pyrogallol.

Item	Substance	Ref.
IUPAC Name ^1^	benzene-1,2,3-triol	[1]
CAS NO. ^2^	87-66-1	
Empirical formula	C_6_H_6_O_3_	
Structural formula	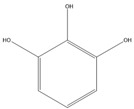	
MW ^3^	126.11 g/mol	
XLogP3 ^4^	0.5	
Physical form	powder	
Solubility	Very soluble in ethanol, diethylether	
Solubility in water	1 g dissolves in 1.7 mL water	
Synonyms	1,2,3-Trihydroxybenzene	

^1^ International Union of Pure and Applied Chemistry; ^2^ Chemical Abstract Service Register Number; ^3^ molecular weight; ^4^ octanol–water partition coefficient.

**Table 2:** Analytical HPLC conditions of pyrogallol.

Item	Condition
Column	Luna 3u C18(2) 100A (150 × 3.00 mm)
Mobile phase	DW (0.1% formic acid): ACN = 95:5
LC condition	Isocratic
Wavelength	200 nm
Column oven temperature	30 °C
Autosampler temperature	4 °C
Flow rate	0.35 mL/min
Running time	6.5 min
Injection volume	10 μL
Retention time	5.4 min

**Table 3:** Accuracy and precision of pyrogallol (n = 5).

Sample	Concentration (μg/mL)	Linearity (r^2^)	Intra ^1^-Day		Inter ^2^-Day	
			Accuracy ^3^ (%)	Precision ^4^ (%)	Accuracy (%)	Precision (%)
SWAB ^5^	0.5	0.99987	103.8	4.9	103.9	4.0
	1.5		100.2	1.5	103.5	2.1
	7.5		99.9	1.3	100.9	1.1
	15		101.6	0.5	102.2	1.1
SC ^6^	0.5	0.99953	106.1	1.6	105.4	1.7
	1.5		102.1	3.3	103.9	2.0
	7.5		101.0	1.1	100.7	0.8
	15		102.4	1.8	103.4	1.1
SKIN ^7^	0.5	0.99948	103.9	2.4	104.9	3.3
	1.5		101.4	3.2	102.3	2.5
	7.5		99.2	1.0	101.2	0.8
	15		101.0	2.0	102.3	1.1
RF ^8^	0.5	0.99939	105.3	6.4	110.1	9.5
	1.5		107.5	1.5	108.2	5.3
	7.5		97.6	3.4	98.0	3.5
	15		92.1	9.0	100.4	6.2

^1^ Repeated five times in one day; ^2^ repeated three times on another day; ^3^ (measured concentration/nominal concentration) × 100 (%); ^4^ (standard deviation/mean) × 100 (%); ^5^ SWAB (30 min and 24 h); ^6^ stratum corneum; ^7^ dermis + epidermis; ^8^ receptor fluid (phosphate-buffered saline (PBS)).

**Table 4:** In vitro dermal absorption of pyrogallol.

	Pyrogallol Content (%) ^1^ (Mean ± Standard Deviation)
SWAB (30 min)	48.4 ± 7.6
SWAB (24 h)	3.9 ± 0.8
^2^ SC	2.3 ± 0.7
^3^ SKIN	10.7 ± 4.7
^4^ RF	15.3 ± 1.6
Total dermal absorption	26.0 ± 3.9
Total recovery	80.5 ± 6.8

^1^ Pyrogallol content was calculated from applied dose of 2.0% pyrogallol; ^2^ SC: stratum corneum; ^3^ SKIN: dermis + epidermis; ^4^ RF: receptor fluid (phosphate-buffered saline (PBS).

**Table 5:** Permeation parameter of 2.0% pyrogallol through excised mini pig skin (mean ± standard deviation).

	Permeation Parameter Js (Equilibrium Flux, μg/cm^2^/h)
Pyrogallol	1.27 ± 0.13

## Data Availability

Not applicable.
